# A First Insight into the Gonad Transcriptome of Hong Kong Catfish (*Clarias fuscus*)

**DOI:** 10.3390/ani11041131

**Published:** 2021-04-15

**Authors:** Xinghua Lin, Dayan Zhou, Xiaomin Zhang, Guangli Li, Yulei Zhang, Cailin Huang, Zhixin Zhang, Changxu Tian

**Affiliations:** 1 Fisheries College, Guangdong Ocean University, Zhanjiang 524088, China; linxinghua97@163.com (X.L.); zhangxiaomin1@stu.gdou.edu.cn (X.Z.); ligl@gdou.edu.cn (G.L.); yuleizhang88@163.com (Y.Z.); 2 Guangdong Research Center on Reproductive Control and Breeding Technology of Indigenous Valuable Fish Species, Guangdong Provincial Engineering Laboratory for Mariculture Organism Breeding, Guangdong Provincial Key Laboratory of Pathogenic Biology and Epidemiology for Aquatic Economic Animals, Guangdong Ocean University, Zhanjiang 524088, China; 3 Guangxi Introduction and Breeding Center of Aquaculture, Nanning 530001, China; magiczdyan@126.com (D.Z.); hcl210305@163.com (C.H.); zhang0474@hotmail.com (Z.Z.); 4 Southern Marine Science and Engineering Guangdong Laboratory, Zhanjiang 524088, China

**Keywords:** full-length transcriptome, *Clarias fuscus*, gonadal development, TGF-β/SMAD pathway, sex-biased genes

## Abstract

**Simple Summary:**

The male Hong Kong catfish (*Clarias fuscus*) grows significantly faster than females, and monocultured males are more commercially available. However, little is known about the molecular regulatory mechanisms of the gonadal development and reproduction process in Hong Kong catfish, limiting the development of monosex cultures. In this study, a high quality transcriptome was constructed from the testes and ovaries of Hong Kong catfish. The regulatory networks of sex-related pathways were explored through Gene Ontology (GO), Kyoto Encyclopedia of Gene and Genome (KEGG) pathway enrichment, and bioinformatics analyses. The results showed that sex-related pathways related to primordial germ cell development, oocyte maturation, gonadal development and steroid biosynthesis were significantly enriched in the gonad transcriptome. This is the first study on the gonad transcriptome of Hong Kong catfish, which provides an important molecular basis for the sex identification and sex-controlled breeding of these catfish.

**Abstract:**

Hong Kong catfish (*Clarias fuscus*) exhibit sexual dimorphism, particularly in body size. Due to the fast growth rate of males, the sexual size dimorphism of Hong Kong catfish has become an economically important trait. However, limited knowledge is known about the molecular mechanisms of sex determination and sex differentiation in this species. In this study, a first de novo transcriptome sequencing analysis of testes and ovaries was performed to identify sex-biased genes in Hong Kong catfish. The results showed that a total of 290,291 circular consensus sequences (CCSs) were obtained, from which 248,408 full-length non-chimeric (FLNC) reads were generated. After non-redundant analysis, a total of 37,305 unigenes were predicted, in which 34,342 unigenes were annotated with multiple public databases. Comparative transcriptomic analysis identified 5750 testis-biased differentially expressed genes (DEGs) and 6991 ovary-biased DEGs. The enrichment analysis showed that DEGs were classified into 783 Gene Ontology (GO) terms and 16 Kyoto Encyclopedia of Gene and Genome (KEGG) pathways. Many DEGs were involved with sex-related GO terms and KEGG pathways, such as oocyte maturation, androgen secretion, gonadal development and steroid biosynthesis pathways. In addition, the expression levels of 23 unigenes were confirmed to validate the transcriptomic data by quantitative real-time polymerase chain reaction (qRT-PCR). This is the first investigation into the transcriptome of Hong Kong catfish testes and ovaries. This study provides an important molecular basis for the sex determination and sex control breeding of Hong Kong catfish.

## 1. Introduction

Sexual dimorphism refers to the differences between males and females of the same species. The sex dimorphism of fish includes the differences in individual size, shape and color, physiology and behavior [1]. For example, the growth rate of half-smooth tongue sole (*Cynoglossus semilae*) female fish is higher than male fish [2], while in Nile tilapia (*Oreochromis niloticus*), the growth rate of male fish is 30% faster than that of female fish [3]. In aquaculture, it is of great economic significance to realize monosex fish culture based on the regulation mechanism of sex determination and differentiation of fish [4]. At present, monosex fish cultures have been conducted with some fish species, such as yellow catfish (*Pelteobagrus fulvidraco*) [5], Nile tilapia [6] and silver barb (*Puntius gonionotus*) [7].

The mechanisms of sex determination and sex differentiation in fish are very complex; they are affected by genetic factors, and closely related to the external environment and its self-endocrine regulation. Genetic sex determination (GSD) is usually determined by single or polygenes located on the sex chromosome [8]. The expression of sex determining genes regulates the signal pathways of sex determination and sex differentiation, inducing the development of primordial gonads to ovaries or testes. Many genes related to sex determination and sex differentiation have been identified in fish, such as doublesex and mab-3 related transcription factor 1 (*dmrt1*), anti-mullerian hormone (*amh*), gonadal soma-derived factor (*gsdf*), forkhead box l2 (*foxl2*), wnt family member 4 (*wnt4*), r-spondin 1 (*rspo1*) and cytochrome p450 family 19 subfamily a member 1a (*cyp19a1a*) [9,10]. Due to the significant differences in the sex determination mechanisms among different fish species, the sex determination genes of teleost fish also show diversity [8,11]. At present, the limited functional genes studied cannot accurately clarify the mechanism of gender regulation. It is necessary to deeply study the law of sex determination and its regulatory mechanisms in different species. Genes involved in sex determination and sex differentiation in fish vary significantly among species. However, no studies comprehensively and accurately elucidate the mechanisms of sex regulation in fish, and intensive studies are needed among different species.

The Hong Kong catfish (*Clarias fuscus*) is a freshwater economic fish cultivated in Southern China [12]. This species has characteristics of strong adaptability, fast growth and delicious meat, which is favored by both farmers and consumers. At the same time, the Hong Kong catfish has obvious sex growth dimorphism. Under the same culture conditions, the growth rate of male fish is significantly faster than female fish at the same age. However, the problem of the high gonadal index of female fish on the market is prominent [13]. Therefore, the monosex fish culture of Hong Kong catfish has important economic significance. Some genes related to sex determination and sex differentiation have been cloned in Hong Kong catfish, such as *dmrt1*, *foxl2* and cytochrome p450 family 19 subfamily a member 1b (*cyp19a1b)*. *dmrt1* is a specific gene expressed in the testis of Hong Kong catfish, which may be closely related to male determination, spermatogenesis and testicular development [14]. *foxl2* may play an important role in the process of gonadal differentiation of Hong Kong catfish [15]. *cyp19a1b* is a factor affecting gonadal differentiation through the hypothalamic pituitary gonadal (HPG) axis in Hong Kong catfish [16]. However, the studies on gonadal development and sex-biased genes of this species are limited, which seriously restricts the promotion and development of the aquaculture industry.

Transcriptome sequencing is a rapid, economical and effective method to detect differences in gene expression, which plays an important role in molecular marker assisted selection [17,18]. Of these, the full-length transcriptome is based on single molecule real-time sequencing (SMRT) technology to obtain complete and high-quality full-length transcripts. This technique can accurately identify the gene information of alternative splicing, gene family and long non-coding RNA (lncRNA). Some transcriptome studies were carried out on amur sturgeon (*Acipenser schrenckii*) [19], pacific white shrimp (*Litopenaeus vannamei*) [20] and golden cuttlefish (*Sepia esculenta*) [21] through a combination of SMRT and Illumina techniques. In this study, a high-quality gonadal transcriptome of Hong Kong catfish was established by the combination of SMRT and Illumina techniques. Comparative transcriptome analysis was conducted for screening sex-biased genes in Hong Kong catfish gonads. This provides a molecular basis for elucidating the mechanism of sex determination and sex differentiation in Hong Kong catfish.

## 2. Materials and Methods

### 2.1. Ethics Statement

All experimental protocols in this study were approved by the Animal Research and Ethics Committee of Guangdong Ocean University (NIH Pub. No.85–23, revised 1996). This study does not involve endangered or protected species.

### 2.2. Sample Collection and RNA Extraction

In this study, Hong Kong catfish (*C. fuscus*) were obtained from the Guangxi Aquatic Products Introduction and Breeding Center, Guangxi, China. Three male (body weight, 213.23 ± 2.90 g) and three female (body weight, 189.97 ± 2.50 g) Hong Kong catfish were collected for gonad sampling. The genders were identified by morphological observation of the gonads. It was shown that the stage IV ovaries were enlarged and appeared long, cystic, full of eggs, and could not flow out of the cloaca [22]. The stage IV testes were inconspicuously branched, the colors were white or light red, the surface folds were obvious, and the blood vessels were thick and many [22,23,24]. According to studies on gonad morphology observation, the ovaries and testes of Hong Kong catfish were in stage IV in this study. Fish were sacrificed by decapitation following deep anesthetization with a eugenol (Macklin, China) bath (1:10,000). Stage IV gonad samples were obtained by dissection, immediately frozen in liquid nitrogen and stored at −80 °C until total RNA extraction.

Total RNA of the gonad was extracted from six fish using RNAiso Plus (Takara, Japan), according to the manufacturer’s instructions. Tissue samples can be fully cleaved in RNAiso Plus. After adding chloroform and centrifugation, the solution was stratified and the supernatant layer (RNA is distributed in the supernatant layer) was collected. Total RNA was recovered by isopropanol precipitation and ethanol cleaning. The RNA sample degradation and contamination was detected by 1% agarose gel electrophoresis. The RNA sample purity was checked by the NanoDrop 2000 (Thermo Scientific, Waltham, MA, USA). The RNA integrity was accurately detected by the Agilent 2100 bioanalyzer (Agilent Technologies, Palo Alto, CA, USA). Total RNA with an RNA integrity number (RIN) score >7 was used for sequencing.

### 2.3. Full-Length Transcriptome Sequencing

The full-length transcriptome library was constructed by mixing RNA samples from three female and three male gonads of Hong Kong catfish. The mixed total RNA was reverse transcribed into complementary DNA (cDNA) by a SMARTer PCR cDNA Synthesis Kit (Takara, Japan), according to the manufacturer’s instructions. The mixture of Total RNA and 3 SMART CDS Primer II A was placed in a centrifuge tube, then incubated at 72 °C for 3 min and treated at 42 °C for 2 min. Then, Master Mix (containing 5× First-Strand Buffer, DTT, dNTP Mix, SMARTer II A Oligonucleotide, RNase Inhibitor and SMARTScribe Reverse Transcriptase) was added to the centrifuge tube to incubate at 42 °C for 1 h, and then heated at 70 °C for 10 min to stop the reaction. The cDNA was amplified by PCR, and the end of the cDNA was repaired. The cDNA was connected with adaptors to obtain the sequencing library (insert size of 1–6 kb). The library was sequenced on a PacBio platform.

The adaptors, low-quality reads and short fragments in the raw reads were filtered to obtain clean reads. The circular consensus sequence (CCS) was obtained through conditional screening (min FullPass = 3 and min Predicted Accuracy = 0.9). Full-length non-chemiric (FLNC) transcripts were determined by searching for the polyA tail signal and the 5′ and 3′ cDNA primers in CCS. Iterative Clustering for Error Correction (ICE) was used to obtain consensus isoforms, which were polished using Quiver. The high-quality transcript was obtained based on the results of consensus isomer polishing (criteria post-correction accuracy > 99%). Cluster Database at High Identity with Tolerance (CD-HIT) [25] was used to remove redundant sequences from high-quality transcripts for obtaining unigenes.

### 2.4. Illumina Transcriptome Sequencing

Six Illumina sequencing libraries (three female ovaries and three male testes of Hong Kong catfish) were constructed using the NEBNext^®^ Ultra™ RNA Library Prep Kit for Illumina^®^ (New England Biolabs, Palo Alto, CA, USA), following the manufacturer’s instructions. The libraries were sequenced on the Illumina Hiseq platform. The clean reads were obtained by removing reads containing adapters, reads containing ploy-N, and low-quality reads from the raw data. At the same time, Q20, Q30 and GC content of the clean data were calculated. The clean reads were mapped to the unigenes from full-length transcriptome by Spliced Transcripts Alignment to a Reference (STAR) [26].

### 2.5. Transcriptome Gene Annotation

To obtain annotation information for the transcripts, the unigenes from the full-length transcriptome predicted the open reading frames (ORFs) using a TransDecoder (https://github.com/TransDecoder/TransDecoder/releases, accessed on 23 December 2020). The unigenes were annotated to the public databases of the National Center for Biotechnology Information (NCBI) Refseq (NR) [27], Gene Ontology (GO) [28], the Kyoto Encyclopedia of Genes and Genomes (KEGG) [29], clusters of orthologous groups for eukaryotic complete genomes, Evolutionary Genealogy of Genes: Non-supervised Orthologous Groups (eggNOG), Swiss-Prot [30] and the Protein families database (Pfam) [31] by Basic Local Alignment Search Tool (BLAST) v2.2.26 (e-value 1 × 10^−5^) [32].

### 2.6. Identification of lncRNAs and Simple Sequence Repeats (SSRs)

lncRNAs and SSRs were identified from the full-length transcriptome of the Hong Kong catfish. Four computational approaches, including the Coding Potential Calculator (CPC) [33], Coding-Non-Coding Index (CNCI) (https://github.com/www-bioinfo-org/CNCI/, accessed on 23 December 2020), Coding Potential Assessment Tool (CPAT) [34], and Pfam [31] were combined to sort non-protein-coding RNA candidates from putative protein-coding RNAs in the transcripts. Putative protein-coding RNAs were filtered out using a minimum length and exon number threshold. Transcripts with lengths greater than 200 nt, having more than two exons, were selected as lncRNA candidates and screened using CPC/CNCI/CPAT/Pfam, which can distinguish the protein-coding genes from the non-coding genes. SSRs were identified in all transcripts (>500 bp) by MISA (http://pgrc.ipk-gatersleben.de/misa/, accessed on 18 January 2021). Two SSR distances less than 100 bp were regarded as compound SSR.

### 2.7. Differential Expression Gene Analysis

The differential expression genes (DEGs) were identified by comparing the Illumina transcriptome of both female and male gonads. The expression level of each transcript was identified using FeatureCounts v1.5.0 [35], to count the number of reads to map each unigene. Then, the fragments per kilobase of transcript per million fragments mapped (FPKM) of each unigene were calculated based on the length of the gene, and reads counts mapped to the unigene. The read counts were adjusted by the edge R program package [36] through one scaling normalized factor for each sequenced library. Differential expression analysis of two samples was performed using the EBSeq R package [37]. The false discovery rate (FDR) was adjusted using the posterior probability DE (PPDE). The FDR < 0.05 and |log_2_(foldchange)| ≥1 were set as the threshold for significant DEGs. GO enrichment analysis of the DEGs was implemented by the GOseq R packages [38] based on Wallenius non-central hyper-geometric distribution. We used KOBAS v2.0 software [39] to test the statistical enrichment of DEGs in KEGG pathways.

### 2.8. Gene Expression Validation

To verify the DEG expression from RNA-seq data, a total of 23 DEGs were randomly selected. The primers of all selected genes were designed by Primer Premier Software v6.0, and are listed in Table S1. A quantitative real-time polymerase chain reaction (qRT-PCR) was performed using TransStart^®^ Green qPCR SuperMix (Transgene, Beijing, China) according to the manufacturer’s instructions on a LightCycler real-time quantitative PCR system (Roche, Indianapolis, IN, USA). The *β-actin* gene was used as an internal reference gene. PCR reactions were performed in triplicate. The relative expression level was measured in terms of threshold cycle value and normalized using the equation 2^−ΔΔCt^.

## 3. Results

### 3.1. Transcriptome Data from the Gonads of Hong Kong Catfish

Based on the PacBio platform, the full-length transcriptome was constructed by SMRT sequencing technology. After quality filtering, 22.25 Gb clean data was obtained from one long-insert (1–6 kb) library. A total of 290,291 circular consensus sequences (CCSs) were obtained through conditional screening (full passes ≥ 3; sequence accuracy > 0.90) of clean data, in which the average of the CCS length was 2506 bp. The CCS contained 5′ and 3′-primers and polyA tail as full-length non-chimeric (FLNC) reads. Consequently, a total of 248,408 (85.57%) CCSs were identified as FLNC reads. The similar sequences (multi-copy transcripts) in the FLNC reads were clustered together. The longest sequence of each cluster was consensus isoform. A total of 69,148 consensus isoforms were obtained, of which 66,958 were high-quality consensus isoforms (sequence accuracy > 99%). The low-quality consensus isoform was corrected by the Illumina transcriptome data, and the 37,305 unigenes were obtained by non-redundant analysis after correction (Table 1).

To identify the sex-biased genes in the gonad tissue of Hong Kong catfish, six cDNA libraries (three male and three female) were generated using the Illumina platform. A total of 144,597,630 clean reads were obtained. The scores of Q20 and Q30 levels ranged from 97.82% to 98.34%, and 94.29% to 95.15%, respectively. The sequence alignment between the Illumina sequencing transcriptome clean reads and full-length transcriptome unigenes was used for differential gene analysis. The results showed that the mapping rate of the female library was higher than 87%, and the rate of the male library was higher than 74% (Table 2).

### 3.2. Transcriptome Annotation

The functions of the unigenes were annotated in the multiple public databases by BLAST (version 2.2.26). A total of 34,342 (92.06%) unigenes were annotated in the public databases. The results showed that 34,221 (91.73%), 26,710 (71.60%), 22,010 (59.00%), 34,144 (91.53%), 29,797 (%), 25,868 (69.34%) and 33,442 (89.64%) unigenes were matched in NR, GO, KEGG, Swiss-Prot, Pfam, KOG (EuKaryotic Orthologous Groups) and eggNOG databases, respectively (Table 3; Figures S1–S4).

### 3.3. Identification of lncRNAs and SSRs

**Figure 1:**
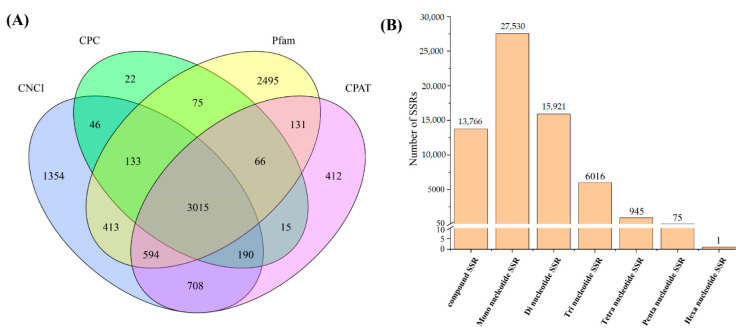
(**A**) Venn diagram of long non-coding RNA (lncRNA) identified from four screening methods. (**B**) Summary of simple sequence repeats of gonad transcriptomes in Hong Kong catfish (*Clarias fuscus*).

Four methods of CPC, CNCI, pfam and CPAT analyses were used to predict lncRNAs. The results showed that 3562, 6453, 6922 and 5131 lncRNAs were predicted by CPC, CNCI, Pfam and CPAT, respectively. A total of 3015 unique transcripts were identified as lncRNAs by four tools (Figure 1A).

A total of 64,254 SSRs were identified from unigenes longer than 500 bp (Figure 1B). Most of the SSRs identified were mono-nucleotide SSR (27,530, 42.85%), followed by the di-nucleotide SSR (15,921, 24.78%), compound SSR (13,766, 21.42%), trinucleotide SSR (6016, 9.36%), tetra-nucleotide SSR (945, 1.47%), penta-nucleotide SSR (75, 0.12%) and hexa-nucleotide SSR (1, 0.0016%).

### 3.4. Identification of Sex-Biased Genes and Enrichment Analysis

**Figure 2:**
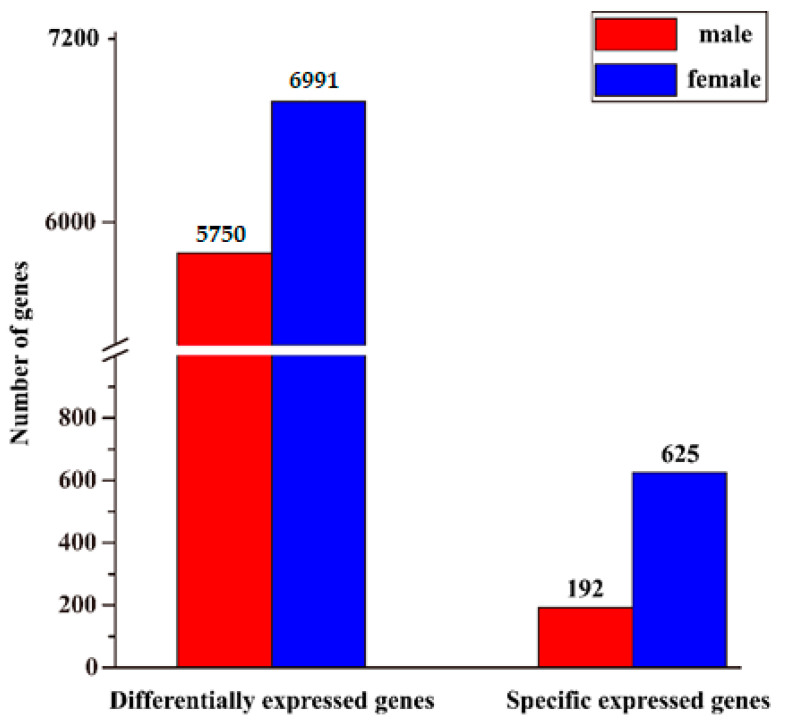
The number of differentially expressed genes (DEGs) and specific expressed genes (SEGs) in testes and ovaries.

Comparative analyses were performed to obtain sex-based genes. A total of 12,741 DEGs were obtained between the testes and ovaries. Of these genes, 5750 unigenes were male-biased DEGs, and 6991 unigenes were female-biased DEGs. In addition, sex-specific genes were detected, including 192 male SEGs and 625 female SEGs (Figure 2, Table S2).

**Figure 3:**
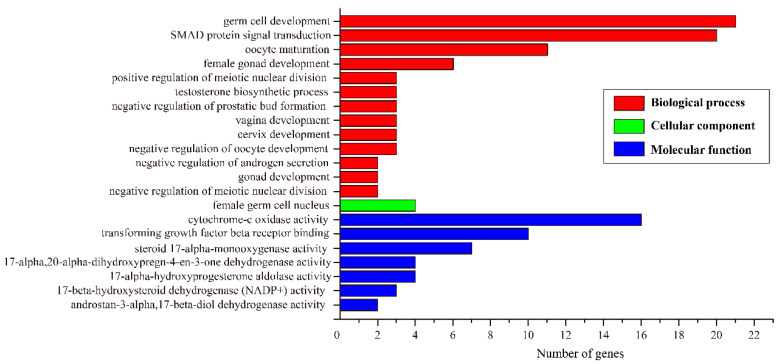
The sex-related gene ontology (GO) terms of differentially expressed genes (DEGs) between testes and ovaries.

To explore genes associated with sexual differentiation and gonadal development, sex-related GO annotation and KEGG pathway analysis was searched for in all DEGs. According to GO enrichment analysis, DEGs were significantly enriched into three main functional categories, including biological process (BP), cellular component (CC), and molecular function (MF), which included 429, 104 and 250 subcategories, respectively (Table S3). In addition, many DEGs were enriched into 21 sex-related GO terms (Figure 3), such as germ cell development (GO:0007281), oocyte maturation (GO:0001556), transforming growth factor beta receptor binding (GO:0005160) and female gonad development (GO:0008585). The DEGs were significantly enriched into 16 signaling pathways via KEGG analysis (Table 4). The sex-related KEGG pathways were associated with gonad development, including the Fanconi anemia pathway (ko03460).

### 3.5. Validation of RNA-seq Results by qRT-PCR

**Figure 4:**
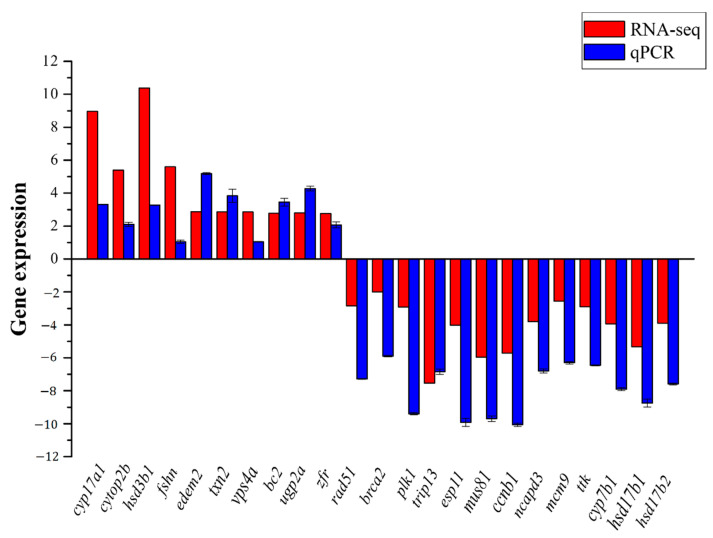
Comparison of expression levels for the 23 significantly-expressed genes (SEGs) using RNA sequencing (RNA-Seq) and a quantitative real-time polymerase chain reaction (qRT-PCR).

To validate the transcriptomic data, a total of 23 genes were randomly used to validate the RNA-seq results (Figure 4). The results showed that the gene expression patterns of the two methods were consistent, indicating the specificity and accuracy of the transcriptome expression analysis.

## 4. Discussion

The Hong Kong catfish is an important freshwater economic fish in Southern China. The growth rates of male Hong Kong catfish are faster than of females, and the gonadal index of females is too high. The male Hong Kong catfish is more valuable than the female in the aquaculture industry. However, there is limited knowledge about the regulatory mechanisms of the reproductive process of Hong Kong catfish, such as sex determination, sex differentiation and gonadal development, affecting the development of the Hong Kong catfish monosex fish culture industry. Therefore, the identification of sex-biased genes and pathways of Hong Kong catfish can provide a theoretical basis for monosex fish culture. In this study, high-quality full-length transcriptome data were obtained from the gonadal tissue of Hong Kong catfish. A total of 290,291 CCSs were obtained, from which 248,408 FLNC reads were generated. After non-redundant analysis, a total of 37,305 unigenes were predicted, in which 34,342 unigenes were annotated with multiple public databases. Comparative transcriptomic analysis identified 5750 testis-biased genes and 6991 ovary-biased genes.

In this study, some of the sex-biased genes were significantly enriched in germ cell development (GO:0007281) GO terms, such as smad family member 5 (*smad5*), lin-28 homolog a (*lin28*) and staufen double-stranded RNA binding protein 1 (*stau1*). The *smad5* and *lin28* were highly expressed in females, while *stau1* was highly expressed in males. In mouse embryos, the deletion of *smad5* resulted in a significant decrease or complete deletion of primordial germ cells (PGCs), indicating that *smad5* is an important factor involved in primordial germ cell generation in mice [40]. *lin28* promotes the expression of the B lymphocyte induced maturation protein (*blimp1*) by inhibiting the maturation of microRNA let-7 to regulate the formation of PGCs [41,42]. The deletion of the *stau1* protein in zebrafish (*Danio rerio*) can lead to abnormal migration of PGCs, indicating that *stau1* plays an important role in the migration of PGCs [43]. *smad5*, *lin28* and *stau1* may play an important role in the formation and migration of primordial germ cells in Hong Kong catfish.

The female-biased genes were significantly enriched in female gonad development (GO:0008585) and oocyte maturation (GO:0001556) GO terms, such as lim homeobox 8 (*lhx8*), folliculogenesis specific BHLH transcription factor (*figla*) and bone morphogenetic protein 15 (*bmp15*). The deletion of *lhx8* leads to the loss of oocytes in the ovaries and the infertility of female mice [44]. *figla* is an effective antagonistic factor, and its overexpression in male tilapia leads to the defection of spermatogenesis, which promotes the development of ovaries [45,46]. *figla* plays a key role in the early ovarian differentiation of zebrafish. In addition, the interaction between *lhx8* and *figla* regulates the growth and differentiation of mouse oocytes [47]. In zebrafish with *bmp15* knockout, gonads are normally developed at an early stage. In later stages, the gonad is reversed from the ovary to the testis and eventually developed into a fertile male. *bmp15* regulates the normal development of female gonads and the maintenance of the female sex [48]. *lhx8*, *figla* and *bmp15* may be involved in ovarian development and oocyte formation in Hong Kong catfish.

Sex determination and sex maintenance are dependent on steroid hormones [49,50], and steroid hormone production is regulated by cytochrome P450 (*cyp*) and hydroxysteroid dehydrogenase (*hsd*) [51,52]. In this study, the GO terms of steroid 17-alpha-monooxygenase activity (GO:0004508), 17-beta-hydroxysteroid dehydrogenase (NADP+) activity (GO:0072582) and androstan-3-alpha,17-beta-diol dehydrogenase activity (GO:0047044) were significantly enriched. Of these, cytochrome p450 family 17 subfamily A member 1 (*cyp17a1*), cytochrome p450 family 11 subfamily C member 1 (*cyp11c1*) and hydroxy-delta-5-steroid dehydrogenase, 3 beta- and steroid delta-isomerase 1 (*hsd3b1*) were highly expressed in males, while hydroxysteroid 17-beta dehydrogenase 1 (*hsd17b1*) and hydroxysteroid 17-beta dehydrogenase 2 (*hsd17b2*) were highly expressed in females. In zebrafish, serum testosterone and 11-ketotestosterone levels decreased significantly in *cyp17a1* knockout individuals, which indicates that *cyp17a1* is involved in androgen synthesis [53,54]. The 11 β-hydroxylase encoded by *cyp11c1* is a key enzyme in the synthesis of 11-ketotestosterone and cortisol (the main androgen and glucocorticoid) in fish [55,56]. *hsd3b1* catalyzes the conversion of pregnenolone to progesterone, 17 α-hydroxyprogesterone to 17 α-hydroxyprogesterone, dehydroepiandrosterone to 4-androstenedione and androstenediol to testosterone. *hsd17b1* and *hsd17b2* catalyze the conversion of low activity 17-ketosteroide, androstenedione and estrone to high activity 17-hydroxysteroids, testosterone and estradiol, respectively [57,58]. *cyp17a1*, *cyp11c1*, *hsd3b1*, *hsd17b1* and *hsd17b2* genes may be involved in the synthesis of gonadal sex hormones in Hong Kong catfish.

The transforming growth factor-β (TGF- β) signal pathway is a large family with many members. This signal pathway mediates the formation of tissues and organs and reproductive development, mainly by regulating the processes of cell growth, proliferation and differentiation [59,60,61,62]. Members of the TGF- β superfamily regulate the transcription of target genes by activating the downstream SMAD pathway [59,63]. The TGF- β signaling pathway is inextricably related to sex determination and sex differentiation of fish. In this study, SMAD protein signal transduction (GO:0060395) and transforming growth factor beta receptor binding (GO:0005160) GO terms were significantly enriched. Of these, transforming growth factor beta 3 (*tgfβ3*), transforming growth factor beta 2 (*tgfβ2*) and smad family member 3a (*smad3a*) were highly expressed in the testes. In contrast, bone morphogenetic protein 15 (*bmp15)*, growth differentiation factor 9 (*gdf9*), bone morphogenetic protein 7a (*bmp7a*), bone morphogenetic protein 2 (*bmp2*), smad family member 2 (*smad2*) and *smad5* were highly expressed in the ovaries. Tgf-β binds to its receptor and activates SMAD2/3 by phosphorylation, which mediates the effect of TGF-β/Activin/Nodal [46]. It is speculated that the high expression levels of *tgfβ3* and *tgfβ2* activate *smad3a* in the testes of Hong Kong catfish. In mouse testes, the deletion of the *smad3* gene delayed the maturation of Sertoli cells and decreased the expression of androgen receptor and androgen-regulated transcripts [64]. *smad3* can regulate the production of follicle-stimulating hormone (FSH) by gonadotropin cells in pituitary glands [65]. FSH binds to its receptor and activates Sertoli cells, participating in spermatogenesis and sperm maturation. Therefore, the high expression of *smad3* in Hong Kong catfish may help to regulate androgen synthesis and spermatogenesis. Some studies have shown that *gdf9* activates *smad2*, rather than *tgf-β* receptors in mouse granulosa cells [66]. Bmp binds to its receptor and activates smad1/5/8, to mediate the effect of BMP/GDF/AMH [61]. It is speculated that the high expression levels of *bmp2* and *bmp7a* activate *smad5*, and the high expression of *gdf9* activates *smad2* in the ovaries of Hong Kong catfish. *smad2* plays an important role in maintaining female fertility and mediating oocyte development [67,68]. *smad2* deletion inhibits the maturation rate of oocytes [69]. *smad5* is not only involved in the formation of PGCs, but is also essential in the process of mouse embryonic development [70]. It is speculated that the high expression levels of *smad2* and *smad5* in the ovaries of Hong Kong catfish may play an important role in oocyte maturation and development. In addition, *bmp15* and *gdf9* may regulate oocyte development through a potential paracrine signal pathway in gibel carp (*Carassius auratus gibelio*) [71]. High expression levels of *bmp15* and *gdf9* were found in the ovaries of Hong Kong catfish, which may be involved in oocyte development. In summary, TGF-β/SMAD pathway plays an important role in the primordial germ cell production, spermatogenesis and oogenesis of Hong Kong catfish.

## 5. Conclusions

This study was the first transcriptomic study of Hong Kong catfish, providing important information for enriching molecular genetic resources. Through the comparison of male and female Hong Kong catfish gonadal transcripts, many sex-biased genes were identified. Through enrichment analysis, several potential genes and pathways related to gonadal development and gametogenesis were screened. These findings will clarify the molecular mechanism of sex determination and gonadal differentiation and contribute to the functional analysis of sex-biased genes in the future.

## Figures and Tables

**Table 1:** Summary of full-length transcriptome sequencing of gonad transcriptomes in Hong Kong catfish (*Clarias fuscus*); CCS—circular consensus sequences; FLNC—full-length non-chimeric.

Item	Full-Length Transcriptome
Number of CCS	290,291
Read bases of CCS	727,649,017
Average read length of CCS	2506
Number of FLNC reads	248,408
Number of consensus isoforms	69,148
Number of high-quality isoforms	66,958
Number of unigenes	37,305

**Table 2:** Summary of the Illumina sequencing of gonad transcriptomes in Hong Kong catfish (*Clarias fuscus*).

Group	Clean Read Number	Clean Base Number	Q30 (%)	Q20 (%)	GC Content (%)	Mapping Rate (%)
Female1	23,589,409	7,063,060,762	95.02	98.27	50.11	88.30
Female2	26,686,421	7,990,222,868	95.01	98.28	49.97	87.40
Female3	23,801,164	7,125,984,844	95.15	98.34	49.94	88.13
Male1	21,172,626	6,329,873,382	94.36	97.82	48.59	74.74
Male2	25,166,743	7,532,229,636	94.29	97.82	48.54	75.58
Male3	24,181,267	7,231,220,526	94.66	97.97	48.14	74.17

**Table 3:** Functional annotation of unigenes from Hong Kong catfish (*Clarias fuscus*) full-length transcriptome; GO—Gene Ontology; KEGG—Kyoto Encyclopedia of Gene and Genome; KOG— EuKaryotic Orthologous Groups; eggNOG—Evolutionary Genealogy of Genes: Non-supervised Orthologous Groups; NR—National Center for Biotechnology Information (NCBI) Refseq.

Annotation Database	Number of Unigenes
GO annotation	26,710
KEGG annotation	22,010
KOG annotation	25,866
Pfam annotation	29,797
Swissprot annotation	34,144
eggNOG annotation	33,442
NR annotation	34,221
All annotated	34,342

**Table 4:** The Kyoto Encyclopedia of Genes and Genomes (KEGG) enrichment of differentially expressed genes (DEGs) between testes and ovaries.

Pathway ID	Pathway Term	*p*-Value
ko03010	Ribosome	1.20 × 10^−14^
ko03460	Fanconi anemia pathway	0.001289
ko00061	Fatty acid biosynthesis	0.002237
ko04110	Cell cycle	0.002476
ko04512	ECM-receptor interaction	0.003232
ko00900	Terpenoid backbone biosynthesis	0.004637
ko03030	DNA replication	0.008156
ko00062	Fatty acid elongation	0.008802
ko00230	Purine metabolism	0.010252
ko00130	Ubiquinone and other terpenoid-quinone biosynthesis	0.010259
ko01212	Fatty acid metabolism	0.014225
ko00670	One carbon pool by folate	0.018081
ko00604	Glycosphingolipid biosynthesis—ganglio series	0.027686
ko00910	Nitrogen metabolism	0.030108
ko04270	Vascular smooth muscle contraction	0.044731
ko00600	Sphingolipid metabolism	0.049852

## Data Availability

The raw data of full-length transcriptome have been submitted to the SRA under accession number SRR13823245. The raw data of Illumina transcriptome have been submitted to the SRA under accession number SRR1382323-SRR13823244 and SRR13823246- SRR13823249.

## Supplementary Materials

The following are available online at https://www.mdpi.com/article/10.3390/ani11041131/s1: Figure S1—homologous species distribution of unigenes annotated in the NR database. Figure S2—annotation of the GO function of the unigenes of *Clarias fuscus*. Figure S3—annotation of the KOG function of the unigenes of *Clarias fuscus*. Figure S4—annotation of the eggNOG function of the unigenes of *Clarias fuscus*. Table S1—quantitative real time PCR (qRT-PCR) primer sequences data. Table S2—differentially expressed genes between testes and ovaries. (|log_2_(fold change) > 1.0| and padj < 0.05). Table S3—Gene Ontology (GO) enrichment of differentially expressed genes between testes and ovaries.
