# Supplementary material for: A First Insight into the Gonad Transcriptome of Hong Kong Catfish (Clarias fuscus)
Source: Animals (Basel). 2021 Apr 15;11(4):1131. doi: 10.3390/ani11041131 (PMC8071282; doi:10.3390/ani11041131)
Supplement: Supplementary file 1 [file animals-11-01131-s001.zip › animals-1157742-supplementary/animals-1157742- Figure S1-S4 for conversion.pdf]

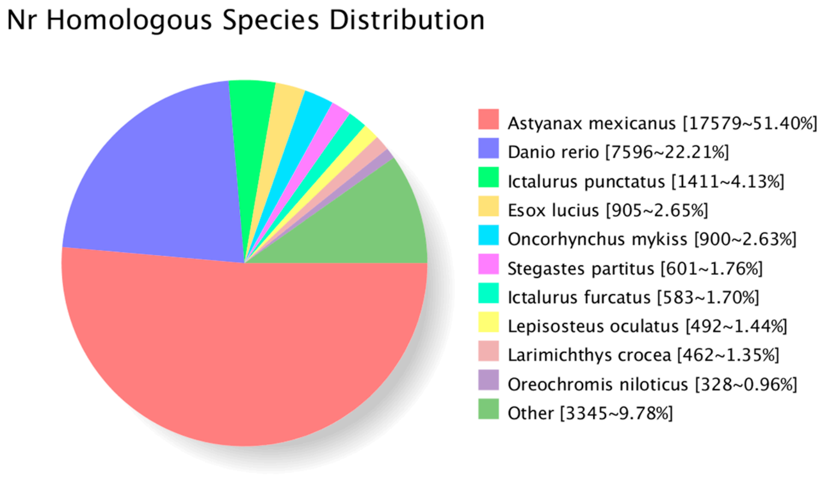

Figure S1. Homologous species distribution of unigenes annotated in the NR database.

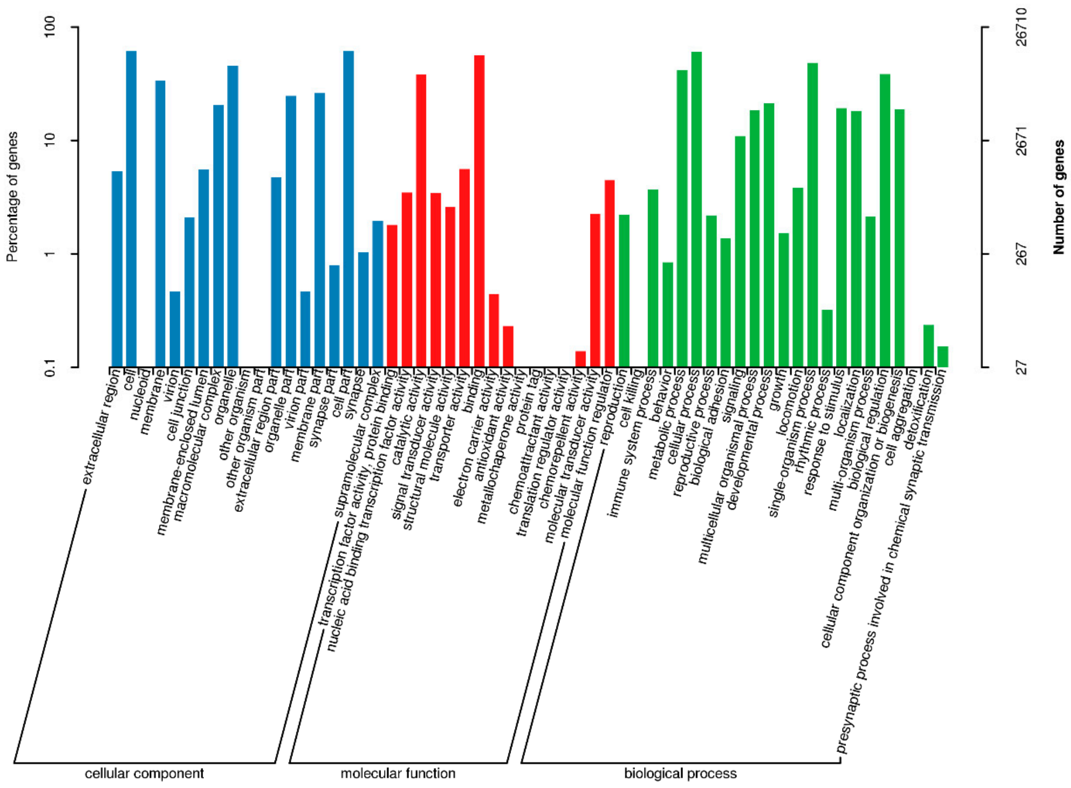

Figure S2. Annotation of the GO function of the unigenes of *Clarias fuscus*.

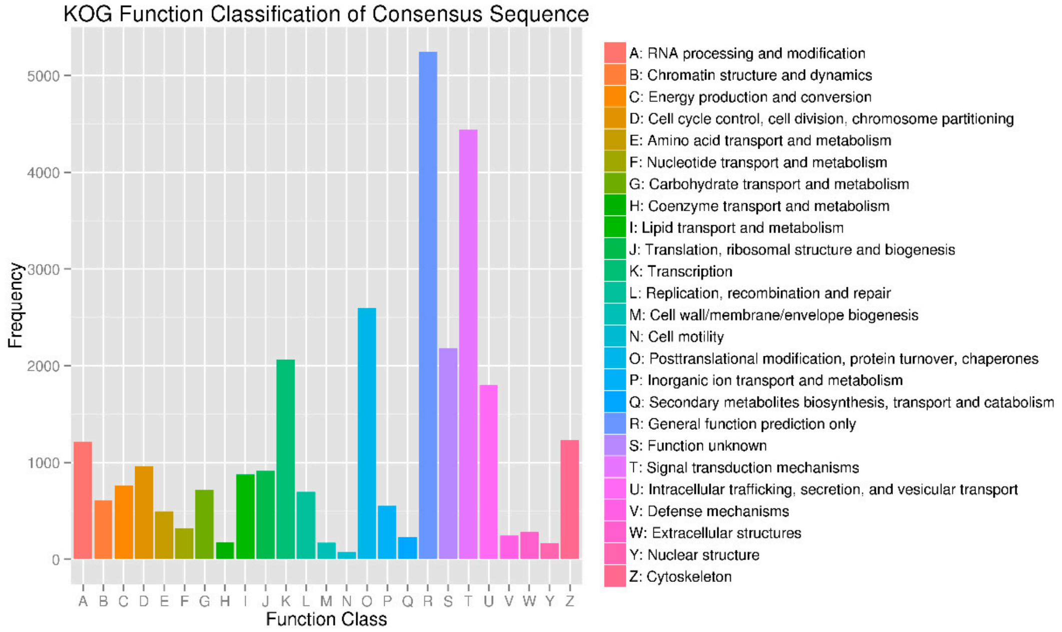

Figure S3. Annotation of the KOG function of the unigenes of *Clarias fuscus*.

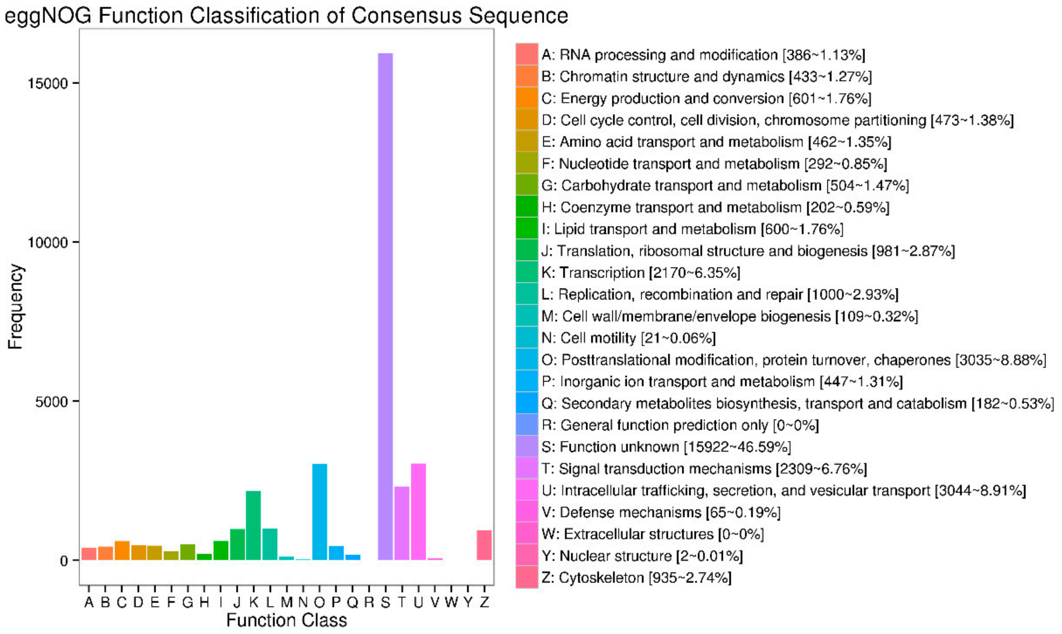

Figure S4. Annotation of the eggNOG function of the unigenes of *Clarias fuscus*.

Table S1–S3 are provided as Excel files.
